# Research on the Response Characteristics of Various Inorganic Scintillators Under High-Dose-Rate Irradiation from Charged Particles

**DOI:** 10.3390/s25175431

**Published:** 2025-09-02

**Authors:** Junyu Hou, Ge Ma, Zhanzu Feng, Weiwei Zhang, Zong Meng, Yuhe Li

**Affiliations:** 1 Department of Precision Instruments, Tsinghua University, Beijing 100084, China; houjy19@mails.tsinghua.edu.cn; 2 Department of Engineering Physics, Tsinghua University, Beijing 100084, China; mg20@mails.tsinghua.edu.cn; 3 School of Nuclear Science and Technology, Lanzhou University, Lanzhou 730000, China; fengzhz2023@lzu.edu.cn; 4 School of Electrical Engineering, Yanshan University, Qinhuangdao 066004, China; zhangww@stumail.ysu.edu.cn (W.Z.); mzysu@ysu.edu.cn (Z.M.)

**Keywords:** inorganic scintillator, response characteristic, high-dose-rate irradiation, radiation detection

## Abstract

With the advent of novel scintillators featuring higher atomic numbers and enhanced radiation hardness, these materials exhibit potential applications under high-dose-rate irradiation. In this work, we systematically compared the photon output characteristics of ten mainstream or emerging inorganic scintillators under high-dose-rate irradiation with low-energy (0.1–1.7 MeV) electrons or protons. Initially, under electron irradiation among ~0.1 to ~50 rad/s, responses exhibited saturation trends to varying degrees, with their variations conforming to the saturation model proposed. However, under proton irradiation among ~5 rad/s to ~150 rad/s, responses exhibited sigmoidal trends due to competition between radiation-induced defects and luminescence centers. Through dynamic derivation of carriers and them, a triple-balance model that demonstrated close agreement with such variations was established. Subsequently, energy-dependent responses under proton irradiation exhibited marked nonlinearity, which were well fitted by Birks’ law, confirming the validity of our measurements. In contrast, electron-induced responses remained nearly linear with increasing energy. Then, after high-dose-rate and prolonged irradiation, BGO revealed highest response degradation, while YAG(Ce) demonstrated most radiation-damage resistance. Moreover, Ce-doped scintillators displayed higher afterglow levels after prolonged irradiation, particularly for YAG(Ce). In summary, these experimental analyses can provide critical guidance for material selection and effective calibration of scintillator detectors operating under high-dose-rate radiation from charged particles.

## 1. Introduction

With the advancement of space science and high-energy physics, the demand for radiation monitoring of charged particles in high-dose-rate environments has increased significantly [1,2]. In scenarios such as Van Allen belts or core areas of next-generation particle accelerators, spacecraft or scientific instruments are exposed to sustained high-dose-rate exposure [3,4], which severely impacts their performance and longevity. Consequently, developing radiation-sensitive materials and their detection systems capable of operating reliably in high-dose-rate environments with long-term stability and superior response characteristics becomes critical for monitoring radiation levels, assessing equipment status, and advancing scientific research.

Over the past three decades, inorganic scintillators have become key materials for radiation detection in space, nuclear technology, and medical applications due to their high detection efficiency, favorable energy resolution, and strong mechanical and environmental adaptability [5]. They can proportionally convert deposited energy of charged particles into photon output, establishing a direct correlation between radiation parameters and photon counts. Microscopically, their scintillation involves a complex multi-stage energy conversion process [6]. Incident charged particles deposit energy, generating hot electrons and holes via inelastic scattering. These hot electron-hole pairs undergo Auger cascades, progressively releasing energy, and thermalized into carriers near the band edges. Under certain conditions, free carriers further form bound excitons, particularly pronounced at high excitation densities. Thermalized carriers and excitons diffuse through the crystal, coupling with luminescence centers through multiple energy transfer pathways. For example, excitons couple to luminescence centers such as rare-earth ions (e.g., Ce^3+^, Pr^3+^), converting them to excited states. Alternatively, luminescence centers can also attain excited states via charge transfer processes or direct carrier capture. Subsequent de-excitation of luminescence centers releases energy through emitting photons, which is called radiative recombination.

However, concurrent non-radiative recombination processes within scintillators might reduce photon output [6,7]. Primarily, irradiation induces the generation and accumulation of various defects. These defects compete with luminescence centers for carrier capture, altering luminescence kinetics and compromising long-term output stability. Next, thermally activated release of carriers trapped by defects contributes to delayed luminescence or afterglow. Under high-dose-rate irradiation, elevated defect concentrations increase the probability of both two processes, significantly changing scintillators’ response characteristics and degrading their long-term performance. Additionally, nonlinear luminescence occurs irrespective of dose rate. At high linear energy transfer (LET), spatially concentrated exciton distributions enhance the probabilities of non-radiative quenching, such as exciton-exciton annihilation or phonon-mediated energy dissipation. Consequently, light yields under heavy-particle irradiation (e.g., low-energy protons) could be substantially lower than those under light-particle irradiation (e.g., electrons). The ionization quenching mechanisms, epitomized by the Birks’ effect, describe this reduction in photon output per unit deposited energy. These effects can also pose severe challenges to response linearity under high-dose-rate and heavy-particle irradiation.

In recent years, inorganic scintillators such as GAGG(Ce) [8], LYSO(Ce) [9], and LuAG(Pr) [10] have been developed and widely adopted owing to their favorable properties. Their high atomic number, high density, and superior radiation resistance make them promising candidates for radiation monitoring in high-dose-rate environments. However, systematic experimental comparisons and supporting theoretical frameworks are still required to scientifically select inorganic scintillators and correctly evaluate their response characteristics and performance limits under such conditions. Previous studies have investigated radiation responses and damage mechanisms in scintillators such as CsI(Tl), BGO, LYSO, and GAGG(Ce) [11,12,13,14]. Nevertheless, the comparison between inorganic scintillators with identical experimental apparatus and irradiation environment remains insufficient, particularly regarding high-dose-rate responses to diverse particle types (e.g., electrons or protons) and varied irradiation parameters (e.g., energy).

To address the above challenges, this study presented a comprehensive investigation of ten widely used or emerging inorganic scintillators (BaF_2_, GAGG(Ce), PWO, YSO(Ce), CWO, LYSO(Ce), YAG(Ce), CsI(Tl), LuAG(Pr), BGO) under well-controlled and high-dose-rate irradiation conditions from charged particles. All scintillators underwent testing with unified processing, packaging, experimental apparatus and environmental conditions, trying to minimize influences from non-intrinsic differences. Building on this foundation, the research focused on four key aspects: (1) quantitative modeling and comparison of dose-rate response behaviors under electron and proton irradiation; (2) analyses of energy response characteristics which highlighted particle diversity in ionization quenching mechanisms and confirmed reliability of the experimental setup and data; (3) evaluation of radiation-induced performance degradation following prolonged high-dose-rate exposure; and (4) systematic assessment of afterglow properties. Compared to previous studies, our approach emphasized direct comparison under unified conditions and model-based analysis, providing robust benchmarks for scintillator selection and detector calibration in challenging radiation environments.

## 2. Experimental Details

### 2.1. Scintillator Selection

In this work, we selected ten mainstream or emerging inorganic scintillators for simultaneous testing, and their compositions and key parameters were shown in Table 1.

Among these scintillators, CsI(Tl) [15], BGO [16], and BaF_2_ [17] have the longest history of applications. The remaining scintillators [8,9,10,18,19,20,21] have emerged and become mainstream over the last two decades. Except for CsI(Tl) and BaF_2_, all others are non-deliquescent and can therefore be conveniently stored and deployed even in complex environments. Given that BaF_2_ and CsI(Tl) are extensively utilized scintillators, employing them in subsequent experiments can enhance the reference value of analytical outcomes. Consequently, despite their slight deliquescence, they were still selected for our study.

### 2.2. Construction of Experimental Apparatus

**Figure 1:**
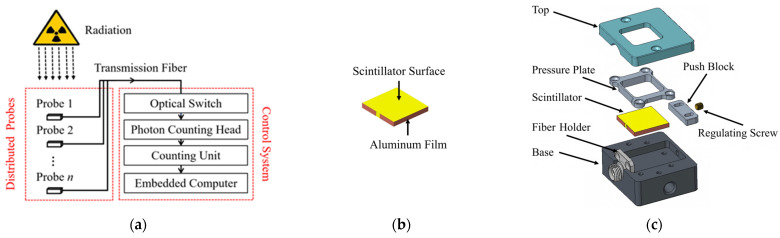
Schematic illustration of experimental apparatus: (**a**) Composition of experimental apparatus; (**b**) Each inorganic scintillator with uncoated windows; (**c**) Structure of each probe.

A compact experimental apparatus was constructed to measure the photon output of these scintillators across varying radiation environments. As illustrated in Figure 1a, the apparatus primarily comprised a control system and multiple replaceable probes.

All ten inorganic scintillators were fabricated into 17 mm (L) × 17 mm (W) × 2 mm (H) plates for deployment in the probes, as shown in Figure 1b. Considering the weak penetration capability of low-energy protons, a 17 mm × 17 mm uncoated surface on each scintillator was directly exposed to radiation. Additionally, a 2 mm × 2 mm uncoated area was reserved at the center of one side facet on each scintillator. This uncoated area interfaced directly with the port of the transmission fibers. All other surfaces were coated by aluminum films with the thickness of ~300 nm for photon reflection. All fabrication and coating processes were completed by Epic Crystal (Kunshan, Jiangsu, China).

The structure of each probe was depicted in Figure 1c. Its primary function was to lock the scintillator while establishing optical coupling with the port of the transmission fiber. The base accommodated the fiber holder and scintillator, maintaining the scintillator plates coplanar with the fiber port relative to the horizontal plane. Rotating the regulating screw drove the push block horizontally to press the scintillator against the fiber holder. The fiber holder fixed the transmission fiber, aligning its port with the uncoated area centered on the scintillator’s side facet. The vertical displacement of the scintillator was constrained by the pressure plate. The top shielded and protected the probe while retaining radiation exposure solely to the scintillators through the window. Each probe measured 35 mm (L) × 30 mm (W) × 20 mm (H). Prior to assembly, both the transmission fiber ports and uncoated areas were cleaned with anhydrous ethanol to minimize the effect of surface contaminants on coupling efficiency.

The transmission fibers were multimode fibers WF50/125/140P from CeramOptec GmbH (Bonn, Germany), cladding with a thickened armored layer to mitigate the impact of scattered particles. Their core/cladding diameters were 50/125 μm, and their numerical aperture was 0.22. Their operational spectral range covered 300–600 nm, encompassing all emission peaks of the selected scintillators.

The control system used an optical switch OSW-1 × 10 from Speed Technology (Shenzhen, Guangdong, China). While its insertion loss (~0.5 dB) reduced transmission efficiency, it allowed sequential measurement of photon outputs from multiple probes with different scintillators under identical irradiation, eliminating interference from radiation parameter variations. By deploying multiple probes with pre-calibrated and identical scintillators, this apparatus also enabled preliminary distributed detection. Transmission fibers routed light to a photon counting head H7828 from Hamamatsu Photonics K.K. (Hamamatsu, Shizuoka, Japan). It contained a head-on photomultiplier tube (PMT), a high-speed photon counting circuit, and a high-voltage power supply circuit. Its spectral operating range (300–650 nm) also covered all emission peaks of the selected scintillators. As its specification indicated, when used together with the counting unit C8855-01 (which was also from Hamamatsu Photonics K.K., Hamamatsu, Shizuoka, Japan), the photon counting head enabled linear counting for photon inputs up to 1.5 × 10^6^ s^−1^. The control system was integrated within an aluminum shell that shielded it from radiation or stray light, with the outer dimensions of 275 mm (L) × 225 mm (W) × 50 mm (H).

Although both the optical attenuation in transmission fibers and the count sensitivity of the photomultiplier tube might vary across wavelengths, they remained relatively consistent within the spectral operating range. Importantly, photon counting failure caused by excessive transmission loss or inadequate sensitivity would not occur at any emission peak of the selected scintillators.

### 2.3. Irradiation Facility and Environment

The irradiation experiments were performed using the high-precision accelerator at the Lanzhou Institute of Physics. The energy of electron or proton radiation could be adjusted within 0–1.7 MeV. The dose rate of electron radiation could be adjusted from roughly 10^4^ rad(Si)/s down to 10^−3^ rad/s, and the dose rate of proton radiation could be adjusted from roughly 10^3^ rad/s down to 1 rad/s. With professional calibration, it exhibited an energy deviation ≤±1% and a current instability ≤±1.5%. Equipped with a Faraday cup and current integrator, it could provide dose or dose-rate data with a measurement error of ≤±1%. And the facility could deliver an emission window of radiation particles with the maximum diameter of ~300 mm. The emission window and the irradiated area were both located in a vacuum chamber, with the vacuum level maintained at ~10^−5^ Pa.

The irradiation room was positioned on the third basement level, with the temperature stable between 15 and 17 °C. The ambient humidity was also controlled at 30–32% throughout the experiment. During the experiment, the irradiation room was strictly maintained free of ambient light. Ten probes were separated by light baffles of certain height to prevent mutual optical interference.

### 2.4. Data Processing

In a defined radiation condition, we recorded real-time photon counts per second during ~10 s for each scintillator. During sequential testing of such ten scintillators via optical switching, both the radiation conditions and experimental environment were kept as stable as possible. The measured responses *N_m_* of selected inorganic scintillators were characterized by the mean photon counts per second, with the unit of s^−1^.

In this experiment, the error bar ${error}_{N_{m}}$ for each measured response *N_m_* primarily represented the fluctuation range in single measurements. These errors originated from three independent components: first, the data variability in multiple measurements arising from factors such as instabilities of the radiation beam, variations in the scintillators’ photon excitation rates, thermal noise fluctuations of the PMT. It was quantified by the standard deviation *σ_N_* of ~10 s response data from measurements. Second, the repeatability deviation of the optical switch, specified by the manufacturer as ≤0.02 dB. This component, denoted as *σ_R_*, could be calculated according to the rules of power attenuation, as shown in Equation (1):

(1) $$10\cdot log\;\frac{{∆N}_{p}}{N_{m}}\leq 0.02\;\to \;{\;\sigma }_{R}={\left({∆N}_{p}\right)}_{max}=10^{\frac{0.02}{10}}\cdot N_{m}\leq 0.5\%\cdot N_{m}$$

where ${∆N}_{p}$ was the maximum output deviation during repeated channel switching with the average output of *N_m_*. Third, a statistical fluctuation component existed for measured response *N_m_*, quantified as $\sigma_{S}=\sqrt{N_{m}}$. Consequently, the error bar ${error}_{N_{m}}$ for certain measured response *N_m_* could be calculated using Equation (2):

(2) $$\pm {error}_{N_{m}}=\pm \sqrt{\sigma_{N}^{2}+\sigma_{R}^{2}+\sigma_{S}^{2}}\leq \pm \sqrt{\sigma_{N}^{2}+{\left(0.5\%\cdot N_{m}\right)}^{2}+N_{m}}$$

However, based on the dead time model [22], there was a conversion caused by PMTs between the measured response *N_m_* and the actual response *N*, as governed by Equation (3):

(3) $$N_{m}=N\cdot e^{-N{\cdot \tau }_{PMT}}\;\;\;\;\;N\in (0,\frac{1}{\tau_{PMT}})$$

where the dead time of the photon-counting PMT was denoted as *τ_PMT_*, and the photon-count loss *L_PMT_* could be calculated using Equation (4):

(4) $$L_{PMT}=\frac{N-N_{m}}{N}\;\;\times 100\%$$

**Figure 2:**
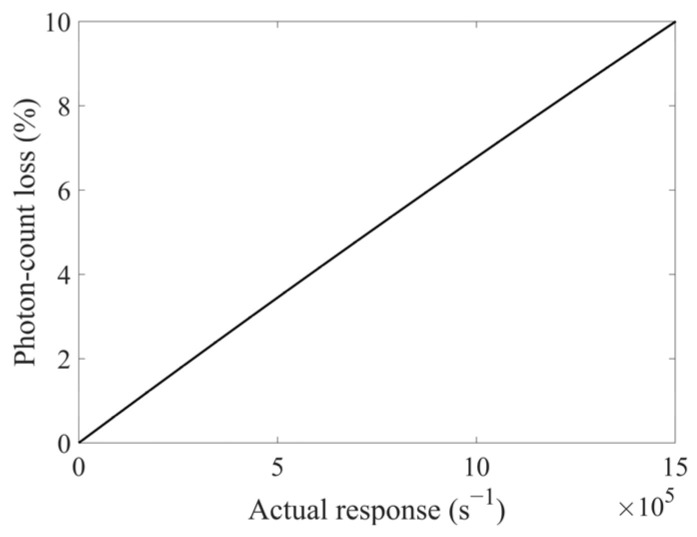
Photon-count loss of the photon counting head H7828 at any actual response *N* in its linear response range.

For the photon counting head H7828 utilized in our experiment, its specification indicated a 10% photon-count loss when *N* reached 1.5 × 10^6^ s^−1^. From this data, we could derive an estimated value *τ′_PMT_* ≈ 70.2 ns, which agreed with *τ_PMT_* = 70  ns in such specification. Using 70.2 ns, the photon-count loss of H7828 at any actual response *N* in its linear response range (0–1.5 × 10^6^ s^−1^) could also be determined, as shown in Figure 2.

Figure 2 demonstrated that the photon count loss increased with higher *N*. This phenomenon introduced nonlinear errors even for the same scintillator when radiation parameters varied. Consequently, using the numerical solver in MATLAB R2023b, all measured responses *N_m_* in our work were converted to actual responses *N* via Equation (3). In subsequent data analysis, the term ‘response’ exclusively referred to the actual responses *N*. In addition, the boundaries of actual responses’ error bars *error_N,upper_* and *error_N,lower_* were similarly derived by, respectively, substituting $N_{m}+{error}_{N_{m}}$ and $N_{m}-{error}_{N_{m}}$ into this same conversion.

The response levels across different scintillators might be influenced by multiple factors, such as the scintillators’ light yield, the coupling efficiency between the scintillator and transmission fiber, the spectral loss in the transmission fiber, and the spectral sensitivity of the PMT. The coupling efficiency was governed not only by inherent properties of the scintillator, such as refractive index and density, but also by positional deviations during coupling. Therefore, the response levels of each scintillator were only reference values measured under this universal experimental apparatus and its corresponding connectivity scheme. For more rigorous research in material science such as assessments of light yields, the experimental apparatus still required respective spectral optimization and compensation for each scintillator. However, it was sufficiently capable of meeting the requirements for scintillators’ comparison. For any given scintillator (and its corresponding channel), these aforementioned factors would remain invariant throughout the experiment and could be treated as a proportionally scaling coefficient. Therefore, in addition to comparing reference response levels, emphasis should also be placed on comparing response variations for the same scintillator under different radiation conditions in our work. This self-referential analysis could reliably reflect the response characteristics of the scintillators.

## 3. Experiment and Discussion

### 3.1. Dose-Rate Response

#### 3.1.1. Electron Experiments

**Figure 3:**
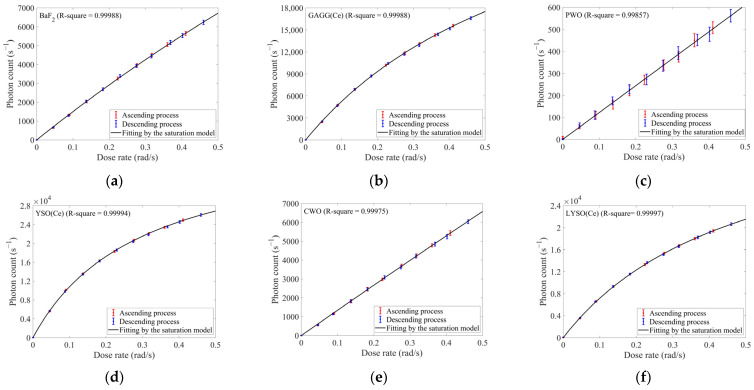

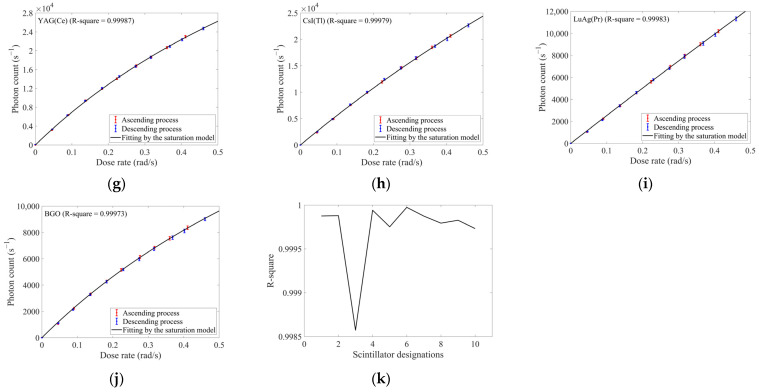
The responses of ten selected inorganic scintillators under 1 MeV electron irradiation with the dose rate ranging from ~0.05 to ~0.5 rad/s (in ~0.05 rad/s increments/decrements): (**a**) BaF_2_; (**b**) GAGG(Ce); (**c**) PWO; (**d**) YSO(Ce); (**e**) CWO; (**f**) LYSO(Ce); (**g**) YAG(Ce); (**h**) CsI(Tl); (**i**) LuAG(Pr); (**j**) BGO; (**k**) R-squares obtained from fitting the dose-rate responses of each scintillator by the saturation model (the correspondence between scintillators and x-axis labels is identical to that in Table 1).

During the experiments on dose-rate responses, both electron and proton energies were set to 1 MeV. For the electron experiments, the dose rate ranged from ~0.05 to ~0.5 rad/s with ~0.05 rad/s increments. After stepwise increasing the dose rate to ~0.5 rad/s, measurements were continued by subsequently decreasing the dose rate in the same steps, to perform a reverse-sequence response test. The responses of ten selected inorganic scintillators were shown in Figure 3.

In Figure 3c, the low response levels of PWO resulted in relatively high fluctuations, leading to wider error bars compared to other scintillators. For the other scintillators, the relative fluctuations were negligible, and their error bars were barely discernible in Figure 3.

Using the responses at the highest dose rate (~0.46 rad/s), ten scintillators were ranked as follows in descending order: YSO(Ce) > YAG(Ce) > CsI(Tl) > LYSO(Ce) > GAGG(Ce) > LuAG(Pr) > BGO > BaF_2_ > CWO > PWO. If dose-rate responses exhibited approximate linearity, higher-ranked scintillators would demonstrate better dose-rate sensitivity in this experiment. However, for YSO(Ce), LYSO(Ce), and GAGG(Ce), near-linear variation in responses persisted only below ~0.2 rad/s. When deploying these scintillators at higher dose rates, calibration for responses’ nonlinearity became essential.

In the ideal case, the dose-rate response should be linear and pass through the origin, meaning that the photon output should be directly proportional to the total deposited energy and independent of irradiation duration. However, the concentration of luminescence centers in scintillators was finite, imposing an upper limit on transient photon conversion. This constraint became particularly pronounced under high-dose-rate irradiation. Assuming a fixed number *m* of luminescence centers in a scintillator, *N_l,u_*(*t*) denoted the number of unexcited luminescence centers at any time *t*, and *N_l,e_*(*t*) represented that in the excited state. They should satisfy Equation (5):

(5) $$N_{l,u}\left(t\right)+N_{l,e}(t)=m$$

Prior to irradiation, *N_l,u_*(0) = *m*, *N_l,e_*(0) = 0. Given a lifetime *τ_l_*, an excited-state center would return to the unexcited state after decay. Under stable irradiation at dose rate $\dot{D}$, the excitation rate of luminescence centers was modeled as linearly proportional to both *R* and *N_l,u_*(*t*). Consequently, upon irradiation commencement, *N_l,e_*(*t*) followed the first-order differential equation given by Equation (6):

(6) $$\frac{dN_{l,e}(t)}{dt}=\beta \dot{D}N_{l,u}(t)-\frac{N_{l,e}(t)}{\tau_{l}}$$

where *β* was the excitation coefficient with units rad^−1^. The first term on the right side represented the generation of excited luminescence centers, while the second term corresponded to the recovery of that. At an excitation-recovery equilibrium, Equation (7) could be derived by $\frac{dN_{l,e}(t)}{dt}=0$:

(7) $$N_{l,e}=\frac{m\beta \tau_{l}\cdot \dot{D}}{1+\beta \tau_{l}\cdot \dot{D}}$$

Furthermore, photons counted for the response originated from the de-excitation processes of excited luminescence centers. Consequently, under steady-state conditions, the response *N* could be expressed by Equation (8):

(8) $$N=\frac{\alpha N_{l,e}}{\tau_{l}}=\frac{\alpha m\beta \cdot \dot{D}}{1+\beta \tau_{l}\cdot \dot{D}}=\frac{am}{\tau_{l}}(1-\frac{1}{1+\beta \tau_{l}\cdot \dot{D}})$$

where *α* denoted the coefficient of conversion efficiency, which comprised quantum efficiency, light collection efficiency, and other factors. The saturation model by Equation (8) demonstrated that when the dose rate was sufficiently small, the response exhibited $N_{linear}\approx \alpha m\beta \cdot \dot{D}$. As the dose rate increased to sufficiently high levels, the response progressively saturated and asymptotically approached a constant value $\frac{am}{\tau_{l}}$. Furthermore, the scintillator with higher *βτ_l_* would display more pronounced saturation trend within identical dose-rate range. We utilized response data acquired during the ascending process of dose rates, and the fitting results were provided in Figure 3 and Table 2.

The response variation of PWO exhibited no significant saturation trend, which might stem from its extremely low carrier generation efficiency under 1 MeV electron irradiation. Across the tested dose-rate range, the carrier density in PWO might remain significantly lower than the density of luminescence centers. Due to the abundant luminescence centers, saturation could be thus avoided. However, this remained a hypothesis based on experimental data, requiring further validation through material science investigations. And this phenomenon also resulted in a slightly lower R square of PWO using the saturation model. In contrast, all other scintillators achieved R squares exceeding 0.99973. Moreover, as shown in Figure 3 and Table 2, scintillators with higher *βτ_l_*, such as GAGG(Ce), YSO(Ce), and LYSO(Ce), exhibited more pronounced curvature on their corresponding response curves within the same dose-rate range. Conversely, PWO which showed greater linearity, had the lowest *βτ_l_*. This consistency indicated that this constant could serve as a quantitative indicator for assessing saturation trends of scintillators’ responses. To sum up, the discussion here validated the efficacy of Equation (8).

Figure 3 clearly showed that for all scintillators, the data acquired during the descending process of dose rates closely followed the fitted curves derived from the data acquired during the ascending process. This hysteresis-free behavior might indicate that no scintillator exhibited radiation-induced degradation in its response during this experiment.

**Figure 4:**
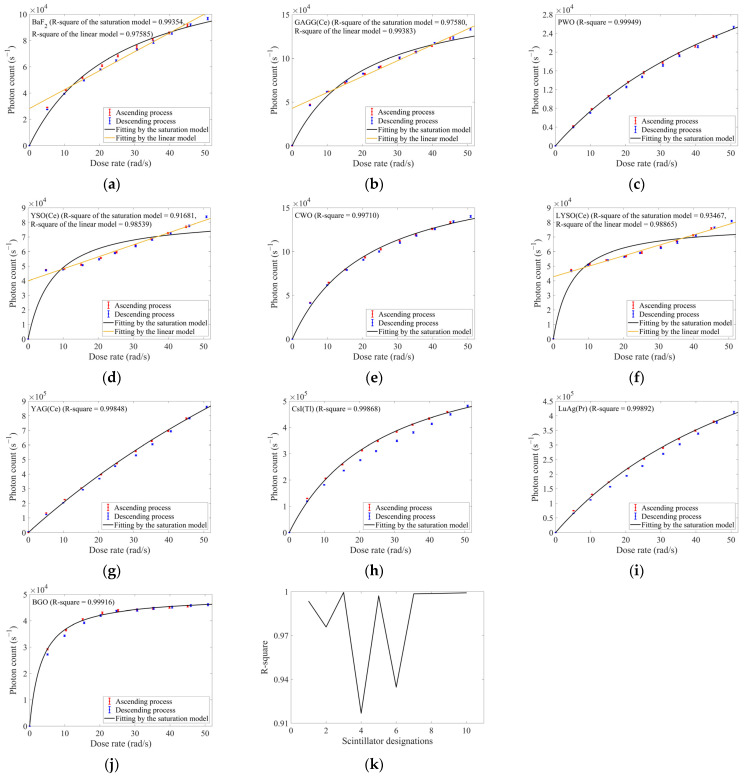
The responses of ten selected inorganic scintillators under 1 MeV electron irradiation with the dose rate ranging from ~5 to ~50 rad/s (in ~5 rad/s increments/decrements): (**a**) BaF_2_; (**b**) GAGG(Ce); (**c**) PWO; (**d**) YSO(Ce); (**e**) CWO; (**f**) LYSO(Ce); (**g**) YAG(Ce); (**h**) CsI(Tl); (**i**) LuAG(Pr); (**j**) BGO; (**k**) R-squares obtained from fitting the dose-rate responses of each scintillator by the saturation model (the correspondence between scintillators and x-axis labels is identical to that in Table 1).

Considering that some scintillators did not exhibit discernible saturation trends within this relatively low dose-rate range, we performed supplemental experiments by electron irradiation at higher dose rates. In this experiment, the dose rate ranged from ~5 to ~50 rad/s with ~5 rad/s increments, and data acquired during both the ascending and descending process of dose rates were also recorded. The responses of ten selected inorganic scintillators were shown in Figure 4. Using response data acquired during the ascending process of dose rates, fitting results of the saturation model were shown in Figure 4 and Table 3.

Using the responses at the highest dose rate (~51 rad/s), ten scintillators were ranked as follows in descending order: YAG(Ce) > CsI(Tl) > LuAG (Pr) > CWO > GAGG(Ce) > BaF_2_ > YSO(Ce) > LYSO(Ce) > BGO > PWO. In both electron irradiation experiments, YAG(Ce) demonstrated outstanding response levels. Moreover, YAG(Ce) maintained acceptable response linearity even across the extended dose-rate range during the second experiment. For electron radiation detection involving dose-rate variations, YAG(Ce) might represent a good material choice. Although CsI(Tl) showed a response level only inferior to YAG(Ce), it exhibited stronger saturation trends in this higher-dose-rate experiment. Furthermore, its slight deliquescence required stricter environmental controls for storage and deployment, constraining its practical applicability. By contrast, LuAG(Pr) exhibited lower response levels than CsI(Tl), but demonstrated milder saturation trends.

The rankings of YSO(Ce) and LYSO(Ce) declined significantly, primarily due to their higher *kτ* in Table 2. Their higher *kτ* would cause pronounced saturation trends even at the lowest dose rate in this experiment. Consequently, the growth rate of their response would not be as rapid as in relatively lower dose-rate ranges. And similarly, because the rapid-rise stage of responses at low dose rates was absent, the data of YSO(Ce), GAGG(Ce) and LYSO(Ce) in this experiment could not characterize their entire evolution of dose-rate responses, resulting in degraded goodness-of-fit for the saturation model. As we added the fitting results of their responses applying linear model in Figure 4 and Table 3, these absences could also explain why their linear fits exhibited elevated goodness-of-fit and significantly elevated intercepts. BaF_2_ also exhibited a similar phenomenon, but its saturation trend was less pronounced for the data within the dose rates of 0–10 rad/s. Except such four scintillators with incomplete response evolution, the saturation models applied to other scintillators both achieved goodness-of-fits exceeding 0.997, further proving the model’s validity. The dose-rate responses of BGO, CWO, and PWO all exhibited a typical evolution featuring an initial rising phase and subsequent saturation trend. Among all scintillators, BGO demonstrated the most pronounced saturation within the dose rates of 25–50 rad/s, as evidenced by its response virtually plateauing.

Figure 4a,h,i revealed distinct inconsistencies between the responses acquired during ascending and descending processes for BaF_2_, CsI(Tl), and LuAG(Pr). Simultaneously, BGO exhibited similar deviations specifically in its non-saturated ranges. In other words, the radiation-induced degradation of responses might occur under persistent and high-dose-rate exposure for these scintillators [6]. Such irradiation might even potentially inflict structural damage within these scintillators [11]. In high-dose-rate applications, this phenomenon warranted careful consideration. And we would conduct prolonged irradiation experiments later to compare response degradation ratios across ten selected scintillators.

In summary, as dose rate increased to high levels, inorganic scintillators exhibited response characteristics that monotonically increased and asymptotically approached saturation under electron irradiation. When dose rate served as the sole independent variable, the concise saturation model we proposed could facilitate effective calibration of these scintillators, enabling reliable *N*-to-$\dot{D}$ conversion. The constant *βτ_l_* in such model could also serve as a quantitative indicator for assessing saturation trends of scintillators’ responses. These analyses would provide practical utility for their selection and deployment in dose-rate detection.

#### 3.1.2. Proton Experiments

**Figure 5:**
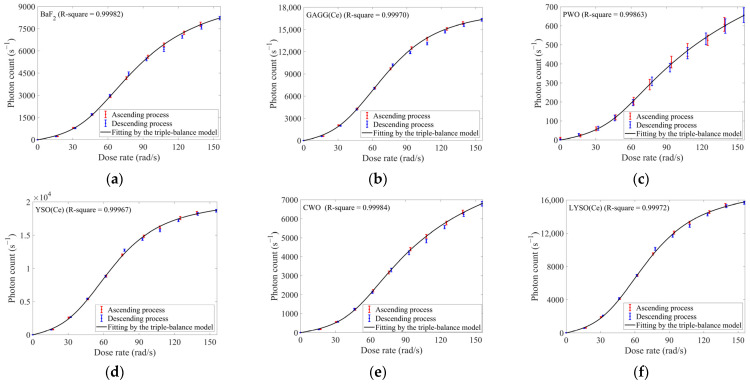

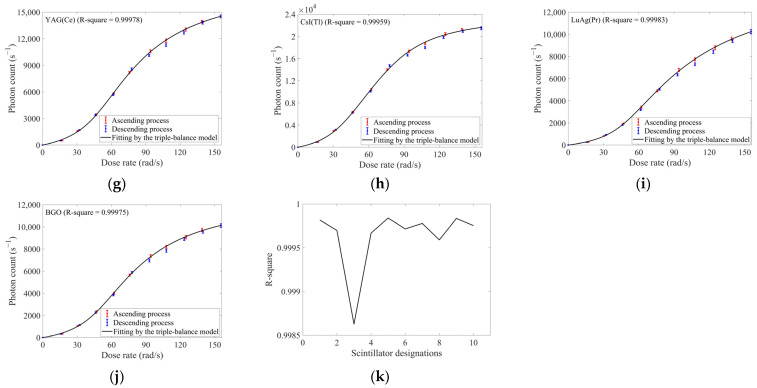
The responses of ten selected inorganic scintillators under 1 MeV proton irradiation with the dose rate ranging from ~15 to ~150 rad/s (in ~15 rad/s increments/decrements): (**a**) BaF_2_; (**b**) GAGG(Ce); (**c**) PWO; (**d**) YSO(Ce); (**e**) CWO; (**f**) LYSO(Ce); (**g**) YAG(Ce); (**h**) CsI(Tl); (**i**) LuAG(Pr); (**j**) BGO; (**k**) R-squares obtained from fitting the dose-rate responses of each scintillator by the triple-balance model (the correspondence between scintillators and x-axis labels is identical to that in Table 1).

For the proton experiments, the dose rate ranged from ~15 to ~150 rad/s with ~15 rad/s increments. Both the ascending and descending processes of dose rates were also conducted. The responses of ten selected inorganic scintillators were shown in Figure 5.

Using the responses at the highest dose rate (~150 rad/s), ten scintillators were ranked as follows in descending order: CsI(Tl) > YSO > GAGG(Ce) > LYSO(Ce) > YAG(Ce) > LuAG(Pr) > BGO > BaF_2_ > CWO > PWO.

Distinct from electron responses, all scintillators exhibited sigmoidal trends in their dose-rate responses under proton irradiation: (1) initial slow rise; (2) accelerated growth at mid-range dose rates; and (3) deceleration at higher dose rates. The last deceleration in phase (3) still arose from response saturation due to limited concentration of luminescence centers, consistent with what was observed in electron irradiation. The slow rise rate (1) and accelerated growth (2) might originate from the competition for carriers between luminescence centers and defect centers [6]. Elevated defect concentrations in irradiated scintillators would increase their probabilities for carrier capture, competing with photon formation processes Given the deep penetration and low LET of electrons, such competition would not macroscopically cause noticeable photon loss due to relatively sparse defect distribution. Conversely, the shallow penetration and high LET of low-energy protons could generate and concentrate more defects in a small region of scintillators. Therefore, the photon output reduction induced by such competition would become more pronounced under low-energy proton irradiation. Consistent with prior analysis, under proton irradiation with lower dose rates (which also meant a lower carrier concentration) in phase (1), there were still many unoccupied defects in scintillators. And these unoccupied defects trapped limited carriers, thereby suppressing both initial photon output and its rise rate. As dose rates increased in phase (2), carrier density rose concurrently. Simultaneously, the carrier-trapping process by defects would also gradually approach saturation. Both effects could enhance the probability of carrier capture by luminescence centers, which was macroscopically characterized by the accelerated growth of photon output.

Given the complex variation in proton dose-rate responses, the model formulation of this experiment should incorporate the kinetics of carriers and competitions with defect centers within the framework of Equation (8). First, under steady irradiation at dose rate $\dot{D}$, we defined *V* as the volume of the photoexcitation area in the scintillator. The generation rate of carriers *G* in this area was given by Equation (9):

(9) $$G=\gamma \dot{D}$$

where *γ* denoted the charge carrier generation coefficient with units of rad^−1^. We assumed that the photoexcitation area contained a dominant type of luminescence center with population *N_l_* and a dominant type of defect center with population *N_d_*. For an individual scintillator, both *N_l_* and *N_d_* were considered constant during a single measurement with short time. Furthermore, the number of excited luminescence centers was denoted *N_l,e_*, so the number of unexcited luminescence centers was *N_l,u_* = *N_l_* − *N_l,e_*. Analogous definitions apply to defect centers: the number of defect centers *N_d,c_* which were in the process of carriers trapping, while unoccupied defect centers *N_d,u_* satisfied *N_d,u_ = N_d_ − N_d,c_*. To describe the process of carrier trapping, we introduced the capture cross-section for luminescence centers *σ_l_* and defect centers *σ_d_*. Letting *v_c_* to represent the average drift velocity of carriers, the macroscopic capture constants for luminescence centers *k_l_* and defect centers *k_d_* could be defined by Equation (10):

(10) $$k_{l}=\frac{\sigma_{l}v_{c}}{V},k_{d}=\frac{\sigma_{d}v_{c}}{V}$$

where the units of both *k_l_* and *k_d_* were s^−1^. Letting *Nc* to denote the number of free carriers, the number of carriers captured per second by unexcited luminescence centers was *k_l_N_l,u_N_c_*, and that captured by unoccupied defect centers was *k_d_N_d,u_N_c_*. Free carriers were depleted through three distinct mechanisms: the aforementioned two capture processes, and intrinsic recombination loss after their average lifetime *τ_c_*. Consequently, a first-order differential equation could be established as Equation (11):

(11) $$\frac{dN_{c}}{dt}=\gamma \dot{D}-k_{l}N_{l,u}N_{c}-k_{d}N_{d,u}N_{c}-\frac{N_{c}}{\tau_{c}}$$

For the excited luminescence centers and occupied defect centers, first-order differential equations could also be established, given, respectively, by Equations (12) and (13):

(12) $$\frac{dN_{l,e}}{dt}=k_{l}N_{l,u}N_{c}-\frac{N_{l,e}}{\tau_{l}}$$

(13) $$\frac{dN_{d,c}}{dt}=k_{d}N_{d,u}N_{c}-\frac{N_{d,c}}{\tau_{d}}$$

where *τ_l_* denoted the average duration of de-excitation on luminescence centers, while *τ_d_* represented the average duration of resetting on defect centers. Defining the occupancy ratios for luminescence centers $x=\frac{N_{l,e}}{N_{l}}$ and defect centers $y=\frac{N_{d,c}}{N_{d}}$, it followed that $N_{l,u}=N_{l}\left(1-x\right)$, $N_{d,u}=N_{d}\left(1-y\right)$. Assuming all three processes described in Equations (11)–(13) reached steady state, we could obtain $\frac{dN_{c}}{dt}=\frac{dN_{l,e}}{dt}=\frac{dN_{d,c}}{dt}=0$. By incorporating the aforementioned steady-state conditions and occupancy ratios into Equations (11)–(13), dose rate *R* could be expressed as a function of *x*, as shown in Equation (14):

(14) $$\dot{D}(x)=A\;x\left[1+\frac{S\left(1-x\right)}{1+\left(P-1\right)x}\right]+\frac{Bx}{1-x}$$

where *A* and *B* were newly introduced fitting coefficients obtained through coefficient consolidation, with $A=\frac{N_{l}}{\gamma \tau_{l}^{2}k_{l}}$ and $B=\frac{1}{\gamma k_{l}\tau_{l}\tau_{c}}$. Additionally, the kinetic ratio between defect-center resetting and luminescence-center de-excitation was defined as $P=\frac{k_{d}\tau_{d}}{k_{l}\tau_{l}}$, while the carrier-capture competition intensity between them was given by $S=\frac{k_{d}N_{d}}{k_{l}N_{l}}$.

Similarly to Equation (8), the response *N* could be expressed by Equation (15):

(15) $$N=\frac{\alpha N_{l}x\left(\dot{D}\right)}{\tau_{l}}=N_{max}\cdot x\left(\dot{D}\right)$$

where α also denoted the coefficient of conversion efficiency, which comprised quantum efficiency, light collection efficiency, and other factors. The saturated photon count was defined as $N_{max}=\frac{\alpha N_{l}}{\tau_{l}}$.

By coupling Equations (14) and (15), we proposed a new model for dose-rate response under proton irradiation, named as the triple-balance model. This model incorporated five fitting coefficients: *A*, *B*, *S*, *P*, and *N*_max_. For any given inorganic scintillator, we could determine the optimal values of these coefficients by some common fitting methods, such as minimizing the residual sum of squares between predicted and actual responses by adjusting the coefficient values. However, due to the complex structure of $\dot{D}$(*x*), we did not provide the explicit analytical expression for *x*($\dot{D}$). Consequently, to predict the response *N* using fitted coefficients, we must first solve Equation (14) numerically (e.g., via bisection or Newton methods) for a solution $x_{i}\in (0,1)$ at a given dose rate $\dot{D}$. Such a solution could be then substituted into Equation (15) to compute response *N*. Both the procedures of coefficient optimization and numerical solution were readily implementable using established libraries in computational environments such as MATLAB R2023b or Python 3.13.3. Despite the model’s complexity increasing computational demands, its practical implementation still remained accessible. We still utilized response data acquired during the ascending process of dose rates, and the fitting results were provided in Figure 5 and Table 4. And except PWO, all other scintillators achieved R squares exceeding 0.99950, which could validate the efficacy of the triple-balance model and its analysis.

Table 4 also indicated that all fitted values of *S* exceeded 5. Given its definition, this suggested that defect centers might capture more carriers than luminescence centers in these scintillators. Consequently, luminescence centers could capture a larger proportion of carriers only when carrier concentrations were sufficiently high and trapping processes by defects gradually reached saturation. This inference aligned with the assumptions in our earlier qualitative interpretation.

Naturally, the triple-balance model was proposed based on some idealized assumptions. In practice, certain scintillators might host multiple dominant luminescence centers [23] or defect centers [11]. Moreover, prolonged irradiation would inevitably alter the population of defects [6]. However, given that increased model complexity risked inducing fitting instability or even failure, we had to implement necessary simplifications to key parameters and their dynamic evolution during modeling.

Within the dose rates of 90–140 rad/s, all scintillators exhibited marginally lower photon counts during the descending process compared to the ascending process. This might stem from radiation-induced response degradation at high dose rates. However, given the remarkable uniformity of these discrepancies across all ten scintillators, this might also be attributed to potential deviations between actual radiation conditions and preset accelerator parameters during experiments.

In summary, due to competitive carrier-capture processes caused by defects, inorganic scintillators exhibited sigmoidal response variation under proton irradiation. This caused greater complexity in the triple-balance model we proposed. When applied to dose-rate monitoring, more experimental data were required to achieve effective calibration for scintillators. However, such model demonstrated high goodness-of-fit to proton dose-rate responses, which was expected to substantially enhance the reliability of calibration.

### 3.2. Energy Response

#### 3.2.1. Electron Experiments

**Figure 6:**
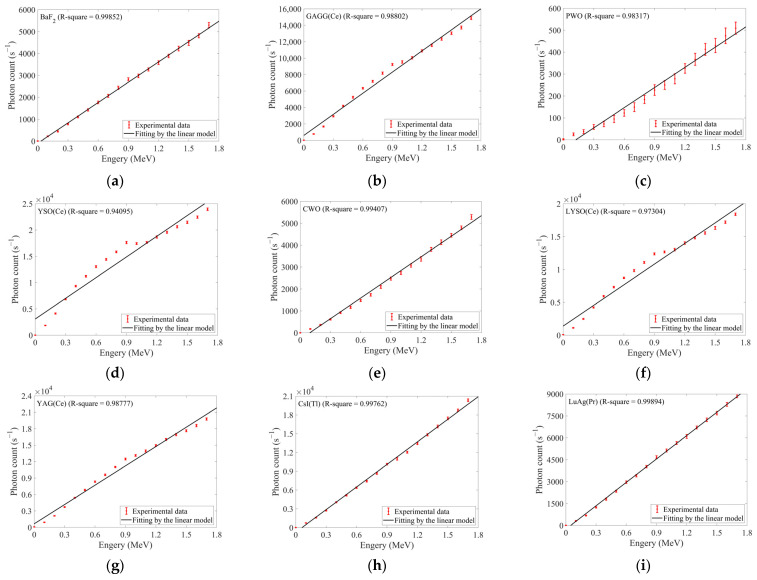

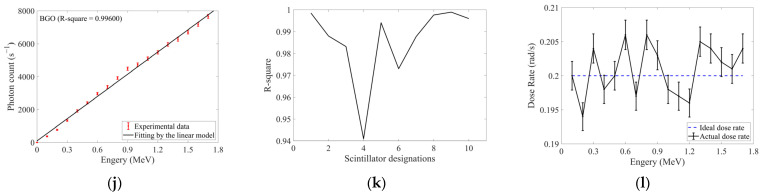
The responses of ten selected inorganic scintillators under 0.2 rad/s electron irradiation with the energy ranging from 0.1 to 1.7 MeV (in 0.1 MeV increments): (**a**) BaF_2_; (**b**) GAGG(Ce); (**c**) PWO; (**d**) YSO(Ce); (**e**) CWO; (**f**) LYSO(Ce); (**g**) YAG(Ce); (**h**) CsI(Tl); (**i**) LuAG(Pr); (**j**) BGO; (**k**) R-squares obtained from fitting the energy responses of each scintillator by a linear model (the correspondence between scintillators and x-axis labels is identical to that in Table 1); (**l**) Actual dose rates at each energy during the experiments.

During the electron energy-response experiments, the dose rate was kept as constant as possible at 0.2 rad/s. The electron energy ranged from 0.1 to 1.7 MeV with 0.1 MeV increments. Given the relatively low upper limit of energy, measurements were conducted solely in ascending order of energies. The responses of ten selected inorganic scintillators were shown in Figure 6, and the fitting results of the linear model were shown in Figure 6 and Table 5.

Using the responses at the highest energy (1.7 MeV), ten scintillators were ranked as follows in descending order: YSO(Ce) > CsI(Tl) > YAG(Ce) > LYSO(Ce) > GAGG(Ce) > LuAG(Pr) > BGO > BaF_2_ ≈ CWO > PWO. This ranking aligned closely with that obtained from the first electron dose-rate experiment. Although CsI(Tl) and YAG(Ce) exchanged positions, their response difference was negligible relative to their levels.

Furthermore, Figure 6 and Table 5 also confirmed near-linear response variations across 0.1–1.7 MeV electron radiation. All scintillators except YSO(Ce) and LYSO(Ce) exhibited R squares exceeding 0.987 for the linear modeling fitted to their electron energy responses. Notably, GAGG(Ce), YSO(Ce), LYSO(Ce), and YAG(Ce) exhibited an anomalous bulge in their energy response curves within 0.8–1.2 MeV, peaking near 0.9 MeV. This phenomenon most likely originated from dose-rate deviations between actual outputs by the accelerator and ideal values. Despite meticulous accelerator tuning to align actual dose rates at each energy with ideal values (0.2 rad/s), actual dose rates consistently resided near ideal values, as depicted in Figure 6l. Each actual dose-rate data in Figure 6l featured an error bar. They comprised two components: (1) measurement errors from the beam integrator *σ_I_* = 1%; (2) a contribution *σ_B_* from beam instability. The ±1.5% beam stability of the accelerator could characterize dose-rate drift during prolonged irradiation (≥~1500 s). Assuming temporally linear dose-rate drift, *σ_B_* within a single measurement cycle (~100 s) could be estimated via Equation (16):

(16) $$\sigma_{B}=1.5\%\cdot \frac{100}{1500}=0.1\%$$

Consequently, the error bar ${error}_{\dot{D}}$ for certain measured dose rate $\dot{D}$ could be calculated using Equation (17):

(17) $${\pm error}_{\dot{D}}=\pm \sqrt{\sigma_{I}^{2}+\sigma_{B}^{2}}=\pm \sqrt{{\left(1\%\cdot \dot{D}\right)}^{2}+{\left(0.1\%\cdot \dot{D}\right)}^{2}}\leq \pm 1.05\%\cdot \dot{D}$$

In the dose-rate response experiments depicted in Figure 3, Figure 4 and Figure 5, data points represented paired measurements of dose rates and corresponding responses. Given their negligible magnitude relative to the extensive x-axis (dose rate) range, error bars along the x-dimension were omitted. Conversely, in Figure 6l, the small scale of the y-axis made these error bars visually discernible. However, even with error bars superimposed, actual dose rates consistently fluctuated around ideal values across energies.

As shown in Figure 6l, dose rates were constrained to 0.2 ± 0.006 rad/s (~±3% deviation) to minimize its effects. Crucially, actual dose rates exceeded the ideal value within 0.8–0.9 MeV but fell below it between 1.0 and 1.2 MeV. Consequently, dose-rate interference should induce initial positive and then negative deviations on ideal photon counts. It manifested as an upward departure from fitted curves followed by re-proximity, with the inflection centered at ~0.9 MeV. For those high-response scintillators, such deviations appeared more pronounced. It also implied that for applications of dose-rate detection, such four scintillators might exhibit superior sensitivity. Concurrently, this anomalous interference significantly degraded the goodness-of-fit for linear models applied to YSO(Ce) and LYSO(Ce).

**Figure 7:**
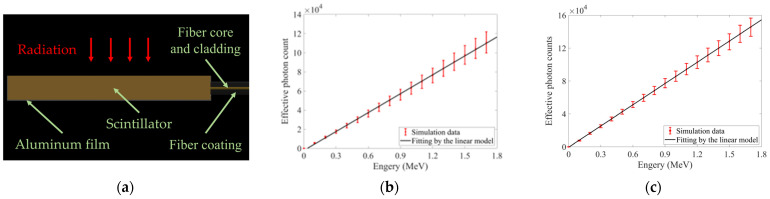
Geant4 simulation of photon generation and propagation in LYSO(Ce) at different energies: (**a**) Coupling structure between the transmission fiber and scintillator; (**b**) Effective photon counts under electron irradiation; (**c**) Effective photon counts under proton irradiation.

Additionally, we have tried to explore other potential causes for the anomalous bulge. A literature review revealed no prior reports of similar anomalies in this energy range. Notably, the transmission fibers were coupled to the center of one side of the scintillator, near the midplane. Particles of different energies penetrated to different depths, potentially causing depth-dependent variations in photon coupling efficiency and introducing nonlinearity into energy responses. To check this hypothesis, we modeled the coupling structure between the transmission fiber and scintillator by Geant4 [24,25,26], as depicted in Figure 7a. To reduce computational load, the fiber core diameter was enlarged to 200 nm and the numerical aperture increased to 0.88, which might proportionally amplify photon coupling probability. However, we stipulated that only photons reaching the fiber core and satisfying the numerical-aperture restriction would be counted as effective. The simulations employed 10^5^ irradiated particles at each energy, and the fiber material was set as SiO_2_. The scintillator was configured as LYSO(Ce) and used its parameters from Table 1. Given the simulation’s objective to investigate the effects of such coupling structure on energy responses, non-uniformity of scintillator responses was not considered during these simulations. We simulated the processes of photon generation and propagation at different energies. Electron and proton simulation results were, respectively, shown in Figure 7b,c.

As shown in Figure 7b, simulated energy responses under electron irradiation displayed near-linear growth in effective photon counts. This indicated that the coupling structure depicted in Figure 7a was unlikely to impose depth-dependent nonlinearity. Collectively, the anomalous bulges in energy-response curves under electron radiation were still most plausibly attributable to dose-rate deviations in accelerator operation.

In summary, although influenced by dose-rate deviations, inorganic scintillators exhibited approximately linear energy responses under low-energy electron irradiation. When using energy as the only variable under high-dose-rate electron irradiation, response-to-energy conversion could be accurately performed after linear calibration.

#### 3.2.2. Proton Experiments

**Figure 8:**
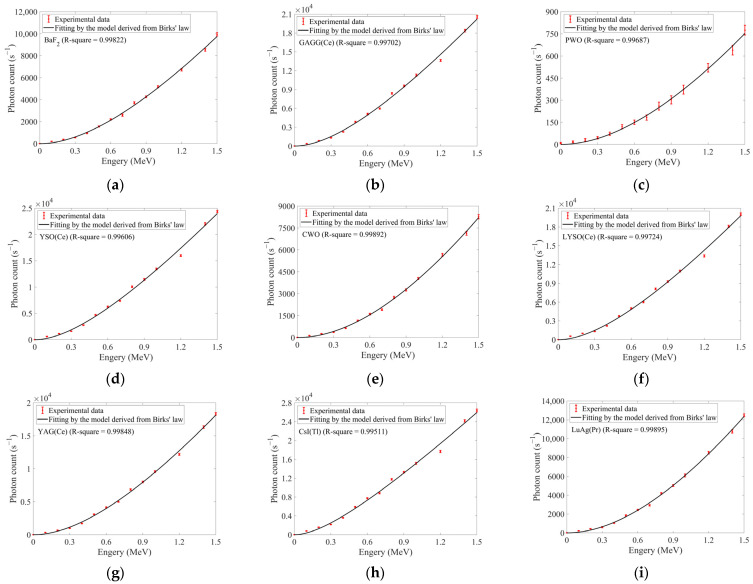

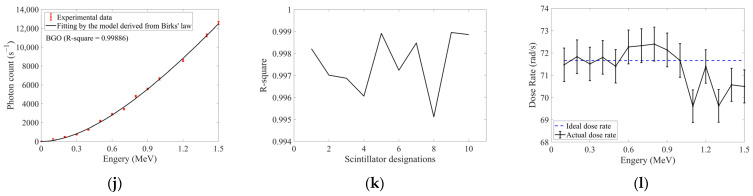
The responses of ten selected inorganic scintillators under ~71.7 rad/s proton irradiation with the energy ranging from 0.1 to 1.5 MeV (in 0.1 MeV increments): (**a**) BaF_2_; (**b**) GAGG(Ce); (**c**) PWO; (**d**) YSO(Ce); (**e**) CWO; (**f**) LYSO(Ce); (**g**) YAG(Ce); (**h**) CsI(Tl); (**i**) LuAG(Pr); (**j**) BGO; (**k**) R-squares obtained from fitting the energy responses of each scintillator by the model derived from Birks’ law (the correspondence between scintillators and x-axis labels is identical to that in Table 1); (**l**) Actual dose rates at each energy during the experiments.

During the proton energy-response experiments, the dose rate was kept as constant as possible at ~71.7 ± 2.1 rad/s (~±3% deviation). Limited by accelerator stability, the proton energy ranged from 0.1 to 1.5 MeV with 0.1 MeV increments. The measurements were conducted solely in ascending process of energies. The responses of ten selected inorganic scintillators were shown in Figure 8.

Figure 8l confirmed dose-rate deviations exceeding the constraint of ±3% deviation at 1.1 MeV and 1.3 MeV. Consequently, response data at these two energies were excluded from the analysis of Figure 8. Using the responses at the highest energy (1.5 MeV), ten scintillators were ranked as follows in descending order: CsI(Tl) > YSO(Ce) > LYSO(Ce) > GAGG(Ce) > YAG(Ce) > BGO > LuAG(Pr) > BaF_2_ > CWO > PWO. This ranking also aligned closely with that obtained from the proton dose-rate experiment.

**Figure 9:**
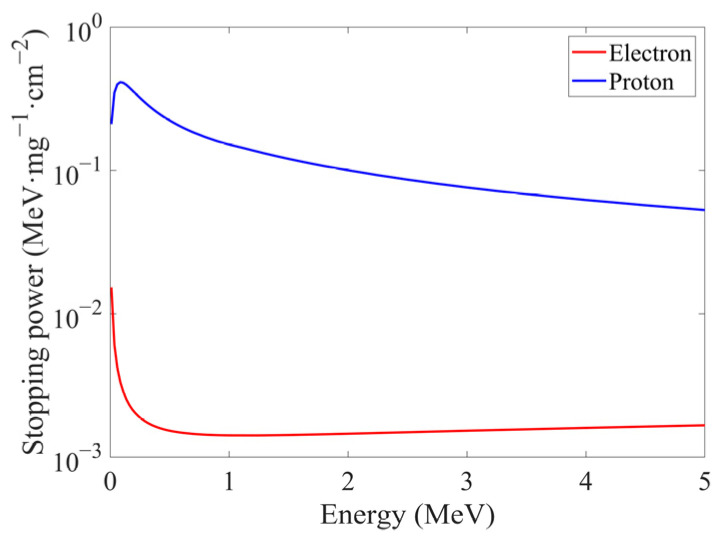
The values of $\frac{dE}{dx}$ under low-energy electrons or protons irradiation in YAG(Ce).

All scintillators in Figure 8 exhibited nonlinear energy responses under proton irradiation. However, Figure 7c showed that energy responses simulated by Geant4 under proton irradiation exhibited near-linear growth in effective photon counts. Therefore, the observed nonlinearity was not attributable to the coupling structures between transmission fibers and scintillators. Such nonlinear phenomenon thus likely related to quenching effects induced by ionization density and enunciated by Birks’ law [7]. Under ionizing radiation (e.g., α particles, protons), the differential form of Birks’ law expressed the relationship between unit-length photon output $\frac{dN}{dx}$ and local stopping power $\frac{dE}{dx}$ at any point along particle trajectories, as expressed in Equation (18):

(18) $$\frac{dN}{dx}=\frac{S_{0}\frac{dE}{dx}\;}{1+K_{B}\frac{dE}{dx}}$$

where *S*_0_ represented the coefficient of scintillation efficiency, and *K_B_* (>0) denoted Birks’ coefficient. Integrating Equation (16) could yield the response *N* of scintillators under radiation with particle energy *E*, as expressed in Equation (19):

(19) $$N=\int_{0}^{E}\frac{S_{0}\;}{1+K_{B}\frac{dE}{dx}}dE\prime$$

where *E*′ denoted the integration variable. In the ideal case where *K_B_* = 0, Equation (19) could simplify to a linear expression *N* = *S*_0_*·E*. And it was obvious that smaller *K_B_* could yield higher linearity between *E* and *N*. The levels of $\frac{dE}{dx}$ for different particles could comparably affect the *E*-*N* linearity in the similar way. Using YAG(Ce) as the irradiated scintillator, electrons exhibited significantly lower $\frac{dE}{dx}$ than protons within the energy ranges of our experiments, as shown in Figure 9. The data of $\frac{dE}{dx}$ under electron irradiation were simulated by SRIM2013 [27], and the data of $\frac{dE}{dx}$ under proton irradiation were excerpted from ESTAR [28].

According to Equation (17), the extremely small $\frac{dE}{dx}$ under electron irradiation could not cause significant nonlinearity in photon count as energy increased. This explained why Birks’ law has been ignored in Section 3.2.1 where a linear model was employed. Experimental data under electron irradiation in Figure 6 and Table 5 also confirmed acceptable linearity of photon outputs. Conversely, protons’ higher $\frac{dE}{dx}$ would produce pronounced nonlinearity, as shown in Figure 8.

To solve Equation (19) analytically, a model for $\frac{dE}{dx}$ was required. Here, a semi-empirical approximation valid for heavy charged particles (e.g., protons) in low-energy ranges [7] was adopted, given by Equation (20):

(20) $$\frac{dE}{dx}(E)\approx \frac{AZ^{2}C}{E}$$

where *C* was the proportionality coefficient, *A* and *Z*, respectively, denoted the mass number and atomic number of the irradiated particle. For protons, *A* = *Z* = 1. Substituting such approximation into Equation (19) could yield an *E*-*N* relationship given by Equation (21):

(21) $$N(E)=\int_{0}^{E}\frac{S_{0}\;}{1+K_{B}\frac{C}{E\prime }}dE^{\prime }={{[S}_{0}E\prime -{S_{0}K}_{B}C\cdot ln(E\prime +K_{B}C)]}_{0}^{E}\;\;\;=S_{0}E-{S_{0}K}_{B}C\cdot ln(1+\frac{E}{K_{B}C})$$

Application of this model to the proton energy responses yielded the results shown in Figure 8 and Table 6. All R-square values exceed 0.995, confirming the efficacy of both above analysis and the model.

In summary, intensified quenching effects resulted in pronounced nonlinearity in the proton energy responses at low energies. The reliability of the experimental setup and data was further confirmed by fitting the measured proton-induced energy responses with Birks’ law, demonstrating good agreement with established theory.

### 3.3. Response Degradation by Prolonged Irradiation

As established in prior sections, prolonged radiation exposure induced persistent generation and accumulation of defect centers in inorganic scintillators. This accumulated radiation-induced damage progressively degraded scintillator performance, manifesting as a reduction in photon output [6,11]. To evaluate the response degradations in ten selected inorganic scintillators after high-dose-rate and high-dose radiation, we conducted a prolonged irradiation experiment.

As analyzed previously, the response variation exhibited certain complexity under proton irradiation, particularly when dose rates or energies changed. To eliminate potential impacts from the selection or variations in radiation parameters on our conclusions, this experiment was only conducted under electron radiation. The irradiation was performed at an electron energy of 1 MeV and a stabilized dose rate of 52.0 ± 0.5 rad/s (~±1% measured deviation), lasting for ~2000 s to accumulate a total dose of ~10^5^ rad. Before and after the experiment, we sequentially measured the responses of all ten scintillators at the same electron energy of 1 MeV. And the data were shown in Table 7.

In Table 7, the degradation ratios of responses *DR* could be calculated from the responses before experiment *N_B_* and the responses after experiment *N_A_* by Equation (22):

(22) $$\;\;\;DR=\frac{N_{B}-N_{A}}{N_{B}}\times 100\%=(1-\frac{N_{A}}{N_{B}})\times 100\%\;$$

Since *N_A_* and *N_B_* were independently measured data, the *DR’s* error bar *error_DR_* could be calculated by Equation (23):

(23) $$\;\;\pm {erro}_{DR}=\pm \sqrt{{\left(\frac{\partial DR}{\partial N_{B}}{error}_{R_{B}}\right)}^{2}+{\left(\frac{\partial DR}{\partial N_{A}}{error}_{R_{A}}\right)}^{2}}\times 100\%\;\;\;\;\;\;\;\;\;\;=\pm \frac{1}{\left|N_{B}\right|}\sqrt{{\left(\frac{N_{A}}{N_{B}}{error}_{N_{B}}\right)}^{2}+{error}_{N_{A}}^{2}}\times 100\%\;$$

where ${error}_{N_{B}}$ was the error bar of *N_B_*, and ${error}_{N_{A}}$ was the error bar of *N_A_*. With the data processing method described in Section 2.4, although the calculated errors for the upper and lower bounds of *N* or *DR* showed slight asymmetry, the discrepancy was negligible. Therefore, the larger value was adopted as the symmetric error for concise presentation in Table 7.

Using the degradation ratios of responses, ten scintillators were ranked as follows in ascending order: YAG(Ce) > CWO ≈ LYSO(Ce) > GAGG(Ce) > LuAG(Pr) > PWO > BaF_2_ > YSO(Ce) > CsI(Tl) > BGO. Lower degradation ratios of responses could indicate higher radiation hardness or resistance for radiation damage. Consequently, YAG(Ce) demonstrated superior radiation resistance in this experiment. LYSO(Ce) and GAGG(Ce) which were also recognized for their radiation resistance [13,14], showed only slightly higher degradation ratios than YAG(Ce). Conversely, BGO which was known to experience significant radiation-induced degradation [29,30], ranked lowest here. These data could provide certain guidance for selecting inorganic scintillators employed in high-dose-rate or high-dose environments.

It should be noted that the dose rate before irradiation was 52.0 rad/s, and 50.3 rad/s after irradiation. This reduction of ~3% could contribute slightly to response degradation. However, this change was consistent for all scintillators and was approximately one order of magnitude smaller than the observed degradation ratios. Thus, the intrinsic properties of the scintillators remained the predominant factor in determining degradation after prolonged irradiation.

**Figure 10:**
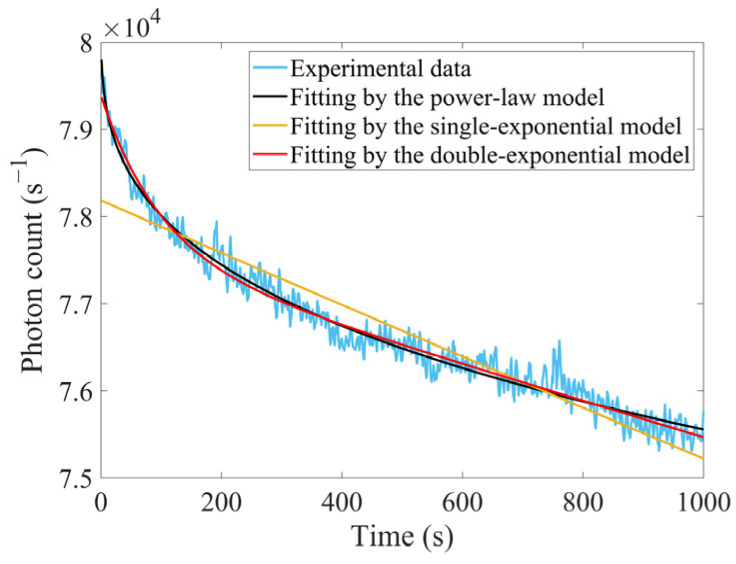
Real-time responses of LYSO(Ce) recorded synchronously with the prolonged irradiation during initial 1000 s.

Commencing synchronously with the prolonged irradiation, the experimental apparatus also recorded 1000 s real-time responses of LYSO(Ce), as shown in Figure 10. As LYSO(Ce) was recognized for its enhanced radiation hardness, its degradation process might offer some representative insights into performance decline mechanisms.

Under radiation exposure, the scintillators’ responses *N* exhibited systematic degradation with accumulated dose *D_T_*. Given the dynamics of defect centers, this trend was commonly approximated by the exponential model as expressed in Equation (24):

(24) $$N=N_{0}\sum_{i=1}^{n}\left(a_{i}\cdot e^{-\frac{D_{T}}{D_{i}}}\right)$$

where *N*_0_ denoted the response at the time *t* = 0, *a_i_* were weighting coefficients. *D_i_* represented dose constants, and *n* specified the number of exponential terms. Equation (24) could be interpreted as an approximate representation of *N* under superimposed effects from multiple defect centers. When *n* = 1, it then simplified to $N=N_{0}e^{-\frac{D_{T}}{D_{0}}}$ [31]. Given the approximately constant dose rate *R* during experiments, substituting $D_{T}=\dot{D}t$ into Equation (24) could yield the expression for response *N* as a function of time *t*. The real-time response of LYSO(Ce) was fitted using both single-exponential (*n* = 1) and double-exponential (*n* = 2) models, with the fitting results presented in Figure 10 and Table 8.

It was observable that the single-exponential model yielded poor goodness-of-fit, whereas the double-exponential model achieved acceptable fitting results. This might suggest that multiple types of defects jointly contributed to response degradation in LYSO(Ce). Additionally, power-law functions were frequently employed as empirical formulas to describe accumulation processes of defect centers in other materials such as quartz [32,33]. Consequently, we also tried to use a power-law model to fit the degenerative responses from LYSO(Ce), as expressed in Equation (25). And as evidenced in Table 8, the power-law model demonstrated comparable goodness-of-fit to the double-exponential model.

(25) $$N=a\cdot t^{b}+c\;\;\;(a<0,\;0<b<1)$$

In this experiment, only the real-time response of LYSO(Ce) was measured under high-dose-rate and prolonged irradiation. Future work would include simultaneous measurements of multiple inorganic scintillators under the same prolonged irradiation to enable comparative analysis of their degradation mechanisms. Advanced characterization techniques would also be necessary to identify the specific types and structures of defect centers responsible for response degradation.

In summary, progressive response degradation occurred in inorganic scintillators during prolonged high-dose-rate irradiation as a result of defect generation and accumulation, compromising long-term detection reliability. When accumulated photon counts were used for total-dose quantification [34], response degradation also introduced additional errors. Our comparison of degradation ratios for ten inorganic scintillators after prolonged electron irradiation provided critical guidance for material selection under such conditions. However, existing empirical models were inadequate for describing degradation kinetics, indicating the need for deeper mechanistic investigations and the development of advanced models.

### 3.4. Afterglows After Prolonged Irradiation

During data processing, the direct current noise was eliminated by algorithms. Under radiation-free conditions before experiments, the responses of all scintillators were measured to be between −20 and 20 s^−1^, with the average of ~0. Additionally, at ~1000 s and ~3500 s after the prolonged electron irradiation in Section 3.3, the responses of all scintillators under radiation-free conditions were recorded. The results were presented in Table 9.

Radiation-free responses measured at both ~1000 s and ~3500 s after the prolonged electron irradiation exhibited positive deviations from zero for all scintillators. This indicated the presence of measurable afterglows in all ten inorganic scintillators tested. As previously mentioned, defect centers in scintillators could capture radiation-induced carriers. These trapped carriers might later be thermally stimulated and released, resulting in delayed luminescence [7]. The delayed luminescence that persisted after the cessation of irradiation was referred to as afterglow.

For all scintillators, the afterglow measured after the second irradiation in Section 3.1.1 and those recorded at ~1000 s after the prolonged irradiation exhibited similar levels. This concordance arose because both experiments involved intense irradiation by high-dose-rate electrons at ~50 rad/s. Additionally, YAG(Ce) exhibited significantly elevated afterglow level (~6500 s^−1^) at ~1000 s after the prolonged irradiation. Although GAGG(Ce), YSO(Ce), LYSO(Ce) and LuAG(Pr) also exhibited certain increase in radiation-free responses, such variations were nearly negligible in comparison with that of YAG(Ce). After a ~3500 s recovery, the afterglow levels of YAG(Ce), LYSO(Ce), GAGG(Ce), and YSO(Ce) exhibited substantial reductions yet remained higher than those of other scintillators. Given the dopants of such four scintillators, Ce-related luminescence centers might be more likely to induce prominent afterglow with relatively slow recovery kinetics. The presence of afterglow might compromise measurement reliability in continuous experiments. For Ce-doped inorganic scintillators, sufficient recovery duration should be allocated between successive applications. In particular, for YAG(Ce) under rapidly fluctuating irradiation, potential masking of low-level responses by intense afterglow must be considered.

Moreover, Table 9 also revealed that only LYSO(Ce) and YAG(Ce) exhibited marginally increased radiation-free responses at ~100 s after proton irradiation in Section 3.1.2. However, the proton-induced afterglow levels were significantly lower, attributable to its weaker penetration capability. The shallow penetration of low-energy protons confined the excitation area to a smaller part of scintillators, thereby reducing the number of carriers contributing to delayed luminescence.

**Figure 11:**
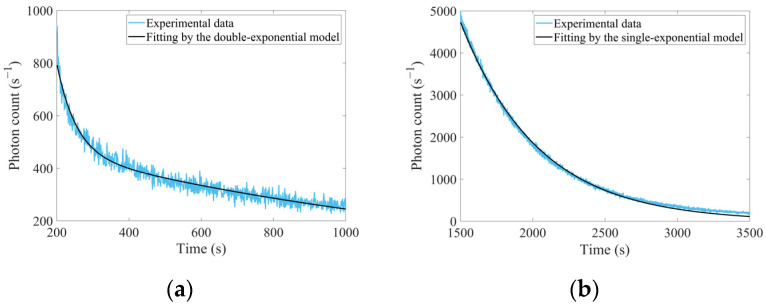
Real-time afterglow levels after the prolonged high-dose-rate electron irradiation: (**a**) Afterglow levels of LYSO(Ce) from ~200 s to ~1000 s after such irradiation; (**b**) Afterglow levels of YAG(Ce) from ~1500 s to ~3500 s after such irradiation.

During experiments, real-time afterglow levels of LYSO(Ce) were recorded from ~200 s to ~1000 s after the prolonged electron irradiation. Furthermore, real-time afterglow levels of YAG(Ce) were also monitored from ~1500 s to ~3500 s after such irradiation. Given that carrier release might originate from multiple defect centers, the afterglow level *I* at any time *t* was commonly modeled as Equation (26) [35]:

(26) $$I=\sum_{i=1}^{n}I_{i}\cdot e^{-\frac{t}{\tau_{i}}}$$

where *I_i_* denoted the initial afterglow level contributed by the corresponding defect center. And *τ_i_* represented the time constants of this defect center, which governed by duration of delayed carrier release. In addition, *n* specified the number of exponential terms, primarily determined by the count of dominant defect species in the scintillator. The afterglow decay processes of both scintillators were fitted using the model described by Equation (26), with results presented in Figure 11 and Table 10.

As evident from Figure 11a, the afterglow levels immediately following the termination of prolonged irradiation significantly exceeded those observed at ~1000 s after irradiation. Regrettably, due to some necessary operations on the accelerator at same time, the measurements of afterglow levels across all scintillators immediately at the termination of irradiation were not conducted. Consequently, synchronous afterglow tracking across multiple scintillators was planned in future experiments.

Table 10 revealed that even the double-exponential model did not adequately fit the afterglow decay process of LYSO(Ce), suggesting again that there might be contributions from multiple types of defects to its evolution. Conversely, despite observable fitting deviations at the end of the curve, the single-exponential model achieved better goodness-of-fit for the afterglow decay process of YAG(Ce). This implied that a single type of defect might play a predominant role in governing afterglow dynamics of YAG(Ce).

In summary, after high-dose-rate electron irradiation, Ce-doped inorganic scintillators exhibited elevated afterglow levels, particularly in YAG(Ce). The slow decay of intense afterglow constrained continuous deployment of such scintillators. In particular, after significant excitation, persistent afterglow might mask low-level responses during detection, reducing effective sensitivity. Given the prevalence of Ce as a dopant, this would impact numerous scintillators’ performance in high-dose-rate environments. The afterglow decay processes were also fitted using exponential models. Collectively, these findings could provide critical guidance for scintillator selection and assessment under high-dose-rate radiation from charged particles.

### 3.5. Discussion on Other Factors

During the experiments, every effort was made to minimize interference from humidity or temperature [36] on scintillators’ responses in this work. The ambient temperature was stringently maintained in 15–17 °C, and the ambient humidity was also controlled at 30–32%. Consequently, their influence on response measurement and data analysis was excluded.

For scintillators, direct optical coupling between photodetectors and scintillator surfaces was commonly employed to maximize coupling efficiency and ensure detection sensitivity [37]. The coupling method implemented in our probe exhibited significantly lower coupling efficiency compared with such approach. However, given that the dose rates in our work were extremely high, this resulted in substantially higher photon output. Maintaining high coupling efficiency would likely drive PMTs into a saturated state. Under such conditions, photon attenuation via optical filters was instead needed to prevent PMT saturation [38]. In contrast, response data confirmed that despite reduced coupling efficiency, our PMT operated on steady state within linear response ranges (photon counts < 10^6^ s^−1^) throughout all experiments. Moreover, although PMTs might be shielded from the major part of radiation doses by scintillators in direct coupling method, progressive radiation-induced degradation of PMTs’ performance remained inevitable under high accumulated doses [39]. Our probe design enabled photon transmission by fibers, allowing PMTs to be housed in radiation-shielded control systems, thereby enhancing operational reliability and longevity. Thus, this probe and its coupling method demonstrated better suitability for high-dose-rate radiation.

Prior to finalizing this design, other probe configurations were also evaluated to achieve acceptable response levels at reduced coupling efficiency. Additional experiments and simulations also revealed that coupling transmission fibers to the scintillators’ bottom surface reduced photon output to <10% of that achieved with side-coupled configurations. As the core objective was to analyze scintillator response variations, detailed results of these additional investigations were omitted here. Future enhancements to detection sensitivity might necessitate probe optimization to improve scintillator-fiber coupling, such as using transmission fibers with a larger core or embedding fibers within grooves in scintillators [40].

Throughout the derivations of models we proposed (such as the saturation model and the triple-balance model), all analyses derived exclusively from fundamental theories of inorganic scintillators, without dependence on material-specific properties. Consequently, these models could exhibit broad applicability across inorganic scintillators. When deployed under similar radiation conditions as specified in this work, they could provide valuable references for response calibration and performance evaluation of other inorganic scintillators.

Since scintillator performance assessment involved multiple types of parameters, identifying a universally optimal material was difficult. For instance, under high-dose-rate irradiation from charged particles, YAG(Ce) demonstrated a higher response level, lower dose-rate saturation trends, and stronger resistance to radiation damage, yet its pronounced afterglow might compromise detection sensitivity during continuous measurements. At dose rates < 0.2 rad/s, scintillators such as GAGG(Ce), YSO(Ce), and LYSO(Ce) exhibited higher dose-rate sensitivity. However, they displayed significant saturation trends at higher dose rates. Alternatively, the experimental results presented in our work could eliminate underperforming candidates in specific applications. For example, BGO showed severe saturation trends and high degradation ratios at dose rates > 25 rad/s, confirming its unsuitability for reliable deployment in high-dose-rate environments. In summary, scintillator selection required the use of practical, application-driven parameter constraints and targeted screening.

## 4. Conclusions

In this work, we systematically compared the response characteristics of ten representative inorganic scintillators (BaF_2_, GAGG(Ce), PWO, YSO(Ce), CWO, LYSO(Ce), YAG(Ce), CsI(Tl), LuAG(Pr), BGO) under high-dose-rate (up to ~50 rad/s for electron and ~150 rad/s for proton) irradiation with low-energy (0.1–1.7 MeV) electrons or protons. A dedicated detection apparatus was developed to ensure the measurements of average photon output per second under identical irradiation conditions.

Initially, electron dose-rate responses exhibited nonlinear variation. Saturation trends remained insignificant across scintillators within the dose rate of ~0.05–0.5 rad/s, while varying degrees of saturation emerged within the dose rate of ~5–50 rad/s. BGO showed the most pronounced saturation, as its response plateaued within the dose rate of 25–50 rad/s. BaF_2_, CsI(Tl), and LuAG(Pr) exhibited hysteresis between the ascending and descending process of dose rates, suggesting radiation-induced degradation. Furthermore, we established a saturation model that accurately fitted the electron dose-rate responses, enabling effective calibration of scintillator response under high-dose-rate detection. The constant *βτ_l_* in this model could also serve as a quantitative indicator for assessing saturation trends. In contrast, as the dose rate of proton irradiation increased, all scintillators exhibited sigmoidal response curves: (1) initial low responses due to defect competition for charge carriers, (2) accelerated response growth as carrier density rose and defect trapping approached saturation, and (3) eventual saturation at higher dose rates limited by available luminescence centers. A triple-balance model incorporating the kinetics of carriers, luminescence centers, and defects was established, providing excellent agreement with proton dose-rate responses and enabling reliable calibration for applications in proton radiation environments.

Subsequently, with dose rate fixed at a constant value, experiments for scintillators’ energy response were conducted within 0.1–1.7 MeV. Under electron irradiation, their responses exhibited near-linear variation trends. The nonlinear fluctuations in the responses of GAGG(Ce), YSO(Ce), LYSO(Ce), and YAG(Ce) were attributed to deviations between actual and ideal dose rates. Under proton irradiation, the observed nonlinearity in the responses due to ionization quenching was well modeled by Birks’ law and stopping-power estimations.

Then, high-dose-rate prolonged electron irradiation (1 MeV, ~52 rad/s, ~2000 s) led to measurable response degradation in all scintillators, attributable to the accumulation of radiation-induced defects. YAG(Ce) demonstrated superior radiation resistance, with LYSO(Ce) and GAGG(Ce) also performing well. In contrast, BGO exhibited the most severe degradation, consistent with prior reports. Existing empirical models failed to accurately describe real-time degradation, indicating the need for further mechanistic and advanced modeling studies.

Finally, afterglow levels of all scintillators at ~1000 s and ~3500 s following prolonged and high-dose-rate electron irradiation were measured. Results indicated that Ce-doped scintillators, especially YAG(Ce), exhibited higher-level and slower-decaying afterglows. Persistent afterglow might mask low responses during continuous operation, reducing detection sensitivity. Furthermore, fitting results of their afterglow decay processes indicated that the dominant types of defects might vary across scintillators. 

In summary, these systematic comparisons comprehensively characterized the response behaviors of ten inorganic scintillators under various irradiation scenarios, with particular emphasis on high-dose-rate environments. These analyses would provide critical guidance for scintillator selection in specific applications. Moreover, the developed saturation model and triple-balance model enabled accurate calibration of scintillator response to dose rate or particle energy, with model constants (e.g., *βτ_l_*) serving as quantitative indicators of response trends. Given that such models derived without dependence on material-specific properties, they also held the potential to be employed for other inorganic scintillators. These advancements would offer evaluation methods for inorganic scintillators and enhance their reliability for effective radiation monitoring under high-dose-rate irradiation from charged particles.

## Figures and Tables

**Table 1:** Ten selected scintillators and their key parameters *.

Label	Scintillator	Composition	Luminescence (nm)	Light Yield (ph/MeV)	Decay Time (ns)	Deliquescence	Refractive Index	Density (g/cm^3^)	Melting Point (°C)
1	BaF_2_	/	300	12,000	650	Slight	1.5	4.89	1280
2	GAGG(Ce)	Gd_3_Al_2_Ga_3_O_12_:Ce	550	54,000	150	No	1.91	6.60	1850
3	PWO	PbWO_4_	530	19,000	30	No	2.16	8.28	1123
4	YSO(Ce)	Y_2_SiO_5_	420	30,000	60	No	1.8	4.45	2000
5	CWO	CdWO_4_	475	13,000	14,000	No	2.3	7.90	1325
6	LYSO(Ce)	(Lu_1−x_Y_x_)_2_SiO_5_:Ce	420	30,000	40	No	1.82	7.25	2050
7	YAG(Ce)	Y_3_Al_5_O_12_:Ce	550	14,000	70	No	1.82	4.56	1970
8	CsI(Tl)	/	550	56,000	1000	Slight	1.79	4.51	626
9	LuAG(Pr)	Lu_3_Al_5_O1_2_:Pr	310	20,000	22	No	1.8	6.7	2010
10	BGO	Bi_4_Ge_3_O_12_	480	8500	300	No	2.15	7.13	1050

* The above data were provided by Epic Crystal (Kunshan, Jiangsu, China). Some data lacked universally recognized standard values and might show slight deviations from data in other sources.

**Table 2:** The fitting results by the saturation model for the responses *N* of ten selected inorganic scintillators under 1 MeV electron irradiation with the dose rate $\dot{D}$ ranging from ~0.05 to ~0.5 rad/s (in ~0.05 rad/s increments).

Label	Scintillator	Fitting Result	R-Square
1	BaF_2_	$N=15476\cdot \dot{D}/\left(1+0.30044\cdot \dot{D}\right)$	0.99988
2	GAGG(Ce)	$N=59848\cdot \dot{D}/\left(1+1.4174\cdot \dot{D}\right)$	0.99988
3	PWO	$N=1222.2\cdot \dot{D}/\left(1+2\times 10^{-14}\cdot \dot{D}\right)$	0.99857
4	YSO(Ce)	$N=143970\cdot \dot{D}/\left(1+3.3567\cdot \dot{D}\right)$	0.99994
5	CWO	$N=13477\cdot \dot{D}/\left(1+0.04743\cdot \dot{D}\right)$	0.99975
6	LYSO(Ce)	$N=86100\cdot \dot{D}/\left(1+1.9974\cdot \dot{D}\right)$	0.99997
7	YAG(Ce)	$N=75595\cdot \dot{D}/\left(1+0.87832\cdot \dot{D}\right)$	0.99987
8	CsI(Tl)	$N=57891\cdot \dot{D}/\left(1+0.36925\cdot \dot{D}\right)$	0.99979
9	LuAG(Pr)	$N=25467\cdot \dot{D}/\left(1+0.06384\cdot \dot{D}\right)$	0.99983
10	BGO	$N=26852\cdot \dot{D}/\left(1+0.78646\cdot \dot{D}\right)$	0.99973

**Table 3:** The fitting results by the saturation model for the responses *N* of ten selected inorganic scintillators under 1 MeV electron irradiation with the dose rate $\dot{D}$ ranging from ~5 to ~50 rad/s (in ~5 rad/s increments).

Label	Scintillator	Fitting Result	R-Square
1	BaF_2_	$N=5524.8\cdot \dot{D}/\left(1+0.03903\cdot \dot{D}\right)$ ($N=1442.2\cdot \dot{D}+28177$)	0.99354 (0.97585)
2	GAGG(Ce)	$N=8429.4\cdot \dot{D}/\left(1+0.04797\cdot \dot{D}\right)$ ($N=1812.1\cdot \dot{D}+42819$)	0.97580 (0.99383)
3	PWO	$N=843.11\cdot \dot{D}/\left(1+0.01407\cdot \dot{D}\right)$	0.99949
4	YSO(Ce)	$N=11778\cdot \dot{D}/\left(1+0.14017\cdot \dot{D}\right)$ ($N=822.91\cdot \dot{D}+39924$)	0.91681 (0.98539)
5	CWO	$N=8944.0\cdot \dot{D}/\left(1+0.04566\cdot \dot{D}\right)$	0.99710
6	LYSO(Ce)	$N=14815\cdot \dot{D}/\left(1+0.18736\cdot \dot{D}\right)$ ($N=718.69\cdot \dot{D}+42704$)	0.93467 (0.98865)
7	YAG(Ce)	$N=21735\cdot \dot{D}/\left(1+0.00582\cdot \dot{D}\right)$	0.99848
8	CsI(Tl)	$N=27113\cdot \dot{D}/\left(1+0.03726\cdot \dot{D}\right)$	0.99868
9	LuAG(Pr)	$N=13844\cdot \dot{D}/\left(1+0.01438\cdot \dot{D}\right)$	0.99892
10	BGO	$N=14169\cdot \dot{D}/\left(1+0.28703\cdot \dot{D}\right)$	0.99916

**Table 4:** The fitting results by the triple-balance model for the responses *N* of ten selected inorganic scintillators under 1 MeV proton irradiation with the dose rate $\dot{D}$ ranging from ~15 to ~150 rad/s (in ~15 rad/s increments).

Label	Scintillator	Fitting Result					R-Square
		*A*	*B*	*S*	*P*	*N*_max_	
1	BaF_2_	101.57	19.744	6.8319	16.493	10,628	0.99982
2	GAGG(Ce)	96.081	9.5889	5.1127	14.062	18,677	0.99970
3	PWO	0.76312	159.09	1613.9	23.308	1471.8	0.99863
4	YSO(Ce)	86.292	10.293	5.0980	12.404	21,340	0.99967
5	CWO	66.556	62.313	15.317	20.184	11,227	0.99984
6	LYSO(Ce)	90.471	11.639	5.7694	14.210	18,296	0.99972
7	YAG(Ce)	77.364	25.956	7.7765	13.593	18,924	0.99978
8	CsI(Tl)	88.647	8.5608	5.2774	14.212	24,177	0.99959
9	LuAG(Pr)	85.369	42.223	11.468	21.155	15,418	0.99983
10	BGO	80.711	24.308	7.8043	14.369	13,097	0.99975

**Table 5:** The fitting results by the linear model for the photon counts *N* of ten selected inorganic scintillators under 0.2 rad/s electron irradiation with the energy *E* ranging from 0.1 to 1.7 MeV (in 0.1 MeV increments).

Label	Scintillator	Fitting Result	R-Square
1	BaF_2_	N=3094.7·E−97.385	0.99852
2	GAGG(Ce)	N=8568.0·E+593.11	0.98802
3	PWO	N=307.34·E−37.882	0.98317
4	YSO(Ce)	N=13120·E+3076.2	0.94095
5	CWO	N=3135.7·E−296.18	0.99407
6	LYSO(Ce)	N=10480·E+1378.6	0.97304
7	YAG(Ce)	N=11759·E+638.00	0.98777
8	CsI(Tl)	N=12028·E−735.39	0.99762
9	LuAG(Pr)	N=5338.6·E−252.22	0.99894
10	BGO	N=4508.7·E+89.617	0.99600

**Table 6:** The fitting results by the model derived from Birks’ law for the responses *N* of ten selected inorganic scintillators under ~71.7 rad/s proton irradiation with the energy *E* ranging from 0.1 to 1.5 MeV and the increments of 0.1 MeV.

Label	Scintillator	Fitting Result	R-Square
1	BaF_2_	N=17633·E−19459·ln(1+E/1.1035)	0.99822
2	GAGG(Ce)	N=27497·E−17143·ln(1+E/0.62344)	0.99702
3	PWO	N=2003.4·E−4085.5·ln(1+E/2.0393)	0.99687
4	YSO(Ce)	N=30895·E−17014·ln(1+E/0.55071)	0.99606
5	CWO	N=26390·E−70001·ln(1+E/2.6525)	0.99892
6	LYSO(Ce)	N=26906·E−16781·ln(1+E/0.62368)	0.99724
7	YAG(Ce)	N=32365·E−34849·ln(1+E/1.0768)	0.99848
8	CsI(Tl)	N=26694·E−7745.8·ln(1+E/0.29017)	0.99511
9	LuAG(Pr)	N=35156·E−78816·ln(1+E/2.2419)	0.99895
10	BGO	N=20905·E−20037·ln(1+E/0.95848)	0.99886

**Table 7:** The responses of ten selected inorganic scintillators under 1 MeV electron radiation before and after the prolonged irradiation experiment.

Label	Scintillator	Response (s^−1^)		Degradation Ratio (%)
		Before Experiment (Dose Rate at 52.0 rad/s)	After Experiment (Dose Rate at 50.3 rad/s)	
1	BaF_2_	102,398 ± 738	73,368 ± 594	28.350 ± 0.714
2	GAGG(Ce)	131,627 ± 926	101,177 ± 1075	23.133 ± 1.030
3	PWO	25,805 ± 268	19,238 ± 210	25.450 ± 1.017
4	YSO(Ce)	81,483 ± 618	51,769 ± 405	36.467 ± 0.590
5	CWO	140,240 ± 907	11,3894 ± 872	18.787 ± 0.801
6	LYSO(Ce)	80,885 ± 571	65,639 ± 576	18.849 ± 0.917
7	YAG(Ce)	881,657 ± 5079	747,109 ± 3927	15.261 ± 0.584
8	CsI(Tl)	538,309 ± 3656	324,998 ± 2787	39.626 ± 0.605
9	LuAG(Pr)	434,704 ± 2724	329,388 ± 2198	24.227 ± 0.634
10	BGO	45,811 ± 357	25,413 ± 217	44.525 ± 0.541

**Table 8:** The fitting results by different models for the real-time responses *N* of LYSO(Ce).

Label	Model	Fitting Result	R-Square
1	Power law	$N=-564.69\cdot t^{0.31012}+80366$	0.97532
2	Single exponent	$N=78185\cdot e^{-\frac{t}{25882}}$	0.89464
3	Double exponent	$N=1805.3\cdot e^{-\frac{t}{96.188}}+77585\cdot e^{-\frac{t}{36125}}$	0.97565

**Table 9:** The afterglow levels of ten selected inorganic scintillators at different times.

Label	Scintillator	Radiation-Free Response (s^−1^)		
		At ~1000 s After the Prolonged Electron Irradiation	At ~3500 s After the Prolonged Electron Irradiation	At ~100 s After the Proton Irradiation
1	BaF_2_	42 ± 9	28 ± 9	0 ± 3
2	GAGG(Ce)	617 ± 37	67 ± 11	2 ± 5
3	PWO	17 ± 7	15 ± 6	−1 ± 4
4	YSO(Ce)	110 ± 14	66 ± 12	2 ± 4
5	CWO	15 ± 6	15 ± 6	−2 ± 3
6	LYSO(Ce)	275 ± 29	147 ± 21	15 ± 9
7	YAG(Ce)	6438 ± 120	167 ± 17	14 ± 6
8	CsI(Tl)	74 ± 15	23 ± 7	−1 ± 3
9	LuAG(Pr)	308 ± 29	28 ± 7	0 ± 5
10	BGO	19 ± 6	15 ± 6	−1 ± 3

**Table 10:** The fitting results by different models for the real-time afterglow levels *I*.

Label	Scintillator	Model	Fitting Result	R-Square
1	LYSO(Ce)	Double exponent	$N=342.04\cdot e^{-\frac{t}{53.575}}+457.17\cdot e^{-\frac{t}{1284.2}}$	0.93537
2	YAG(Ce)	Single exponent	$N=4800.6\cdot e^{-\frac{t}{519.63}}$	0.99808

## Data Availability

The raw data supporting the conclusions of this article will be made available by the authors on request.

## Abbreviations

The following abbreviations are used in this manuscript:

LET	Linear Energy Transfer
PMT	Photomultiplier Tube
NA	Numerical Aperture
